# An Attempt at Quantifying Factors that Affect Efficiency in the Management of Solid Waste Produced by Commercial Businesses in the City of Tshwane, South Africa

**DOI:** 10.1155/2012/165353

**Published:** 2012-11-12

**Authors:** Yohannes Worku, Mammo Muchie

**Affiliations:** ^1^Department of Civil Engineering, Faculty of Engineering, Tshwane University of Technology, Private Bag X680, Pretoria 0001, South Africa; ^2^Faculty of Economics and Finance, Institute for Economic Research on Innovation (IERI), Tshwane University of Technology, Private Bag X680, Pretoria 0001, South Africa

## Abstract

*Objective*. The objective was to investigate factors that affect the efficient management of solid waste produced by commercial businesses operating in the city of Pretoria, South Africa. *Methods*. Data was gathered from 1,034 businesses. Efficiency in solid waste management was assessed by using a structural time-based model designed for evaluating efficiency as a function of the length of time required to manage waste. Data analysis was performed using statistical procedures such as frequency tables, Pearson's chi-square tests of association, and binary logistic regression analysis. Odds ratios estimated from logistic regression analysis were used for identifying key factors that affect efficiency in the proper disposal of waste. *Results*. The study showed that 857 of the 1,034 businesses selected for the study (83%) were found to be efficient enough with regards to the proper collection and disposal of solid waste. Based on odds ratios estimated from binary logistic regression analysis, efficiency in the proper management of solid waste was significantly influenced by 4 predictor variables. These 4 influential predictor variables are lack of adherence to waste management regulations, wrong perception, failure to provide customers with enough trash cans, and operation of businesses by employed managers, in a decreasing order of importance.

## 1. Introduction

The city of Tshwane is the capital of South Africa. According to Statistics South Africa, the population size of the city of Tshwane in 2011 was roughly equal to 3 million. The city is home to over 5,000 businesses out of which 1,603 businesses operate within the Central Business District of Tshwane, known as Pretoria [1]. According to the annual report issued by the city of Tshwane Metropolitan Municipality for the budget year 2010-2011, about 1,734,295 tons of solid waste are collected each year from businesses operating in the city [2]. The solid waste produced by businesses in the city includes trash or garbage such as wood, product packaging, empty bottles, used tyres and car parts, and cans, garden refuse, furniture, clothing, leftover food, newspapers, wires, grease, appliances, paint, pieces of metal, broken containers, sheet metal, used medicine, and the like. These businesses produce massive volumes of solid and liquid waste on a daily basis. Taxi ranks, bus stations, open flea markets, food outlets, and small businesses located in Pretoria are synonymous with litter, uncontrolled solid and liquid waste, as well as lack of capacity in the efficient management of waste. The annual report released by the city of Tshwane Metropolitan Municipality for the year 2011 shows that massive waste is accumulated during strike action by municipal workers responsible for the removal of waste from households and businesses [2]. The collection, disposal, and processing of waste produced by businesses and households are regulated by legislative policies set out and enforced by the Municipality of Tshwane and the South African National Department of Environmental Affairs and Tourism [2, 3]. The use of an integrated municipal solid waste management system [4–7] has been shown to be essential for improving overall efficiency in municipal waste management in almost all developed nations of the world. In order for an integrated waste management system to perform efficiently, all relevant stakeholders of the waste chain must play a mutually collaborative role in the collection, disposal, processing and management of waste. A review of the relevant literature shows that such a measure is essential for reducing the overall cost of waste management, and for the protection of the environment [8–10]. Overall efficiency in the management of waste depends on adherence to municipal acts on waste disposal and management [11–13]. Research work carried out in several developing nations of the world has shown that health education on environmental sanitation and primary health care [14], socioeconomic incentives [15], as well as a strict enforcement of municipal bylaws [16] are all needed for ensuring overall environmental cleanliness and the efficient management of waste in metropolitan cities such as Tshwane.

The study was conducted against the background of a host of factors that are well known to undermine overall efficiency in the management of solid waste in almost all metropolitan cities of the developing world. These background factors are lack of infrastructural capacity required for the efficient management and processing of solid waste [2, 3, 17, 18], poor awareness about the benefits of proper waste management [19], lack of socioeconomic incentives to stakeholders relevant to the waste management chain [20], failure to adequately utilize modern waste management and processing technology [21, 22], failure to vigorously enforce municipal bylaws [2, 3], and the absence of an integrated waste management system in Tshwane [23, 24]. The study aims to identify and quantify the key predictors of inefficiency in the management of solid waste in the city of Tshwane. With a view to ensure optimal efficiency in the management of solid waste, this research work will assess the degree to which municipal bylaws and regulations that are relevant to solid waste management are adhered to by businesses conducting business in the city of Tshwane. The bylaws and regulations are relevant to all stages of the waste management chain (sorting of waste at source, collection, treatment, disposal, and processing). The model considers 4 categories of solid waste (municipal, organic, plastics, and electronic) and is designed for realizing a sustainable and optimally efficient solid waste management system for the city of Tshwane. The model is accompanied by a comprehensive monitoring and evaluation plan that could be used for assessing the current status-quo, and for assessing and evaluating efficiency in waste management at each stage of the waste management chain. Norms and standards set out by the South African National Department of Environment and Tourism are used for benchmarking [4].

Municipal bylaws that are relevant to waste management are not enforced with full vigour. Although more than 25% of small and medium-sized businesses as well as informal traders operating in the city of Tshwane generate waste in the course of conducting routine business [2, 3, 17], the pace at which such waste is properly collected and disposed of by municipal workers employed by the city of Tshwane is significantly lower than the pace at which waste is generated. This situation is exacerbated at times of civil action carried out by municipal workers responsible for routine waste collection and disposal. Taxi ranks, the various sources of solid and liquid waste and pollution, bus stations, liquor stores, petrol stations, schools, shops, open markets, garages, and so forth are well known contributors for the generation of high volume of solid and liquid waste in the CBD of Pretoria. There is room for improvement of the current level of efficiency in the management of waste produced by the various sources of solid and liquid waste and pollution. In terms of the strategic plan adopted by the South African Department of Environmental Affairs and Tourism (DEAT) to improve efficiency in the management of waste, the various sources of solid and liquid waste and pollution are a major stakeholder, and no progress can be made unless otherwise the various sources of solid and liquid waste and pollution are involved in the implementation of the plan drawn up by the DEAT [4, 17].

There is a significant accumulation of solid waste especially in townships such as Mamelodi and Marabastad, and at the various taxi ranks in and around the city of Pretoria. There is a shortage of essential facilities such as trash cans, seats, shelters, public toilets, and clean tap water at taxi ranks and public gathering arenas. Some of the various sources of solid and liquid waste and pollution do not have properly functioning toilets and washrooms although the proper functioning of such facilities is an essential requirement for business. The various sources of solid and liquid waste and pollution, taxi ranks, and public gathering arenas are often characterized by bad smell and a large accumulation of solid and liquid waste. Such places are conducive for the spread of communicable diseases such as food poisoning and diarrhoea unless they are controlled and managed efficiently by the CTMM. Not enough is known so far about the extent to which efficiency in waste disposal can be improved in view of the fact that there is lack of empirical evidence in this area of research interest. Not enough research has been done in the CBD of Pretoria to assess and identify factors that affect efficiency in proper waste disposal. There is a shortage of empirical evidence based on a properly designed multivariate modelling in areas related to the quantification of factors that contribute to poor environmental sanitation and the accumulation of solid and liquid waste in the CBD of Pretoria. Low level of awareness in areas related to recycling, classification of waste at source and composting plays a major role in compounding the current lack of efficiency in the efficient utilization of resources such as broken bottles, empty cans, used goods, plastic bags, and so forth. Far from being put to economic use, broken bottles are harming pedestrians and school children in addition to polluting the environment and decreasing the beauty of the city in the eyes of visitors and residents. Ratepayers are disgusted with the level of incompetence, inefficiency, and indifference. Some irresponsible inhabitants of the city and visitors who travel to the city from outside do not have adequate respect for environmental sanitation and demonstrate total disregard for cleanliness of the streets of the city. Such irresponsible inhabitants and visitors often throw away rubbish on the streets. Examples of such rubbish are beer bottles, empty cans, and used food packages. Empty bottles break into harmful pieces as they are thrown onto the streets from moving taxis and private cars. There is a need to have irresponsible inhabitants and visitors educated and disciplined so that they show respect for environmental sanitation and cleanliness. Experience drawn from large municipalities in Sweden, the United States, and Kenya shows that these deeply ingrained problems could be addressed adequately by enforcing the relevant municipal bylaws with vigour [18], by promoting health and environmental education, and by providing socioeconomic incentives to stakeholders and role-players in the waste management chain [19].

A review of the literature shows that in cities as big as Tshwane, modern waste treatment technologies such as composting, incineration, landfills, recycling, and window composting should be used for solid waste management [20–23]. Modern solid waste management techniques such as landfills, incineration, recycling, biological processing, energy recovery, radio frequency identification tags, global positioning system tracking integrated software packages, rear vision cameras, and the like are used in cities such as Geneva and Stockholm [18]. Examples of advanced solid waste treatment technologies that are suitable for the city are anaerobic digestion, ethanol production, biodrying, gasification, in-vessel composting, mechanical biological treatment, mechanical heat treatment, plasma arc waste disposal, pyrolysis, sewage treatment, and tunnel composting are also highly appropriate technologies for the city [19, 20]. In the city of Tshwane, the management of solid waste involves collection, transportation, processing or disposal, management, and monitoring of waste materials. The South African Department of Environmental Affairs and Tourism [4] promotes the use of modern technologies and evaluation techniques that are necessary for the efficient management of waste although advanced technologies that are used for waste management and evaluation are poorly utilized in South African cities including the City of Tshwane [2].

Globally, diverse methods have been used for improving efficiency in the collection and disposal of solid waste. The most commonly used methods are designed for ensuring efficiency in solid waste management and are outlined in the UN-Habitat's Third Global Report of 2010 [21]. In the world's cleanest cities, the following methods are used: integrated sustainable waste management, the enforcement of municipal bylaws, the promotion of primary health care services and environmental sanitation, the promotion of health education on proper waste management, financial sustainability, institutional framework, good governance, community based campaigns of awareness, the provision of incentives for good performance in the collection of solid waste, process flow diagrams, as well as monitoring and evaluation programmes designed for the assessment of municipal service delivery programmes [22–25].

In this study, assessment of efficiency in waste management was made by using the classic structural time-based model constructed by Tchobanoglous et al. [26] as well as adherence to municipal bylaws. The city of Tshwane uses the model for assessing overall efficiency in the collection of solid waste by municipal service providers. The model is suitable for assessing efficiency in the collection of solid waste from fixed containers that are used for depositing solid waste by businesses and ordinary residents. The model assesses overall efficiency as a function of time taken for the management of waste.

The aim of this research is to identify and quantify factors that affect overall efficiency in the management of solid waste generated by businesses that operate in the city of Tshwane. Based on data collected from a random sample of *n* = 1,034 businesses operating in the city of Tshwane, the research article aims to:

1. assess the current state of solid waste management with regards to type of wastes and quantities of waste generated,
2. identify key factors that affect efficiency in the management of solid waste, and
3. assess the degree to which businesses adhere to norms and standards that are recommended for businesses in terms of solid waste disposal.

## 2. Methods

### 2.1. Data

Data was collected in 2010 from a random sample of 1,034 businesses operating in the city of Tshwane. Eligible businesses were identified based on a sampling frame belonging to Statistics South Africa [1]. Managers and owners of the businesses as well as employees were asked a total of 89 questions related to the type of business they were operating, the type and volume of waste generated, how waste was collected, sorted out, disposed of and managed, the extent of cleanliness and sanitation at the workplace, their socioeconomic and demographic characteristics, as well as their personal opinion on the benefits of the proper management of waste.

### 2.2. Statistical Analyses

Efficiency in solid waste management was assessed by using the model proposed by Tchobanoglous et al. [26] for estimating the median time required for the collection of solid waste from the businesses that took part in the study. The model measures efficiency in the collection of solid waste from fixed locations and containers based on the mathematical expression shown below:

(1) $$\begin{array}{c} E=1-A+B. \end{array}$$

In (1), *A* denotes an off-route factor, and *B* is a measure of off-route activity time by individuals collecting waste. In cases where *A* = *B*, the collection of solid waste becomes perfectly efficient. The larger the difference between *A* and *B*, the less efficient becomes the waste collection procedure. The time required per trip is given by the expression shown below:

(2) $$\begin{array}{c} t=\frac{t_{1}+t_{2}+Y}{1-A}. \end{array}$$

In (2), *t* is the time required for waste collection from a fixed site per trip, *t*_1_ is equal to pick up time of container at the site of collection, *t*_2_ is equal to on-site time per trip, *Y* denotes the total haul time during waste collection, and *X* denotes the average round-trip haul distance per trip.

Since the relationship between *Y* and *X* follows simple linear regression, we have

(3) $$\begin{array}{c} Y=\beta_{0}+\beta X. \end{array}$$

In (3), *β*_0_ is the constant term in the simple linear regression of *Y* on *X*, and *β* is the regression coefficient in the simple linear regression of *Y* on *X*. In cases where waste is collected from fixed sites, the time required to collect waste per trip follows a stationary model, and is expressed by the expression shown below:

(4) $$\begin{array}{c} t_{1}=P+Q+R. \end{array}$$

In (4), *P* is the time taken to pick up a container that is full of solid waste; *Q* is the time taken to unload an empty container; *R* is the time taken for driving from one container to the next container.

The number of trips per day is given by the expression shown below:

(5) $$\begin{array}{c} n=Y\left(1-A\right)-\frac{\left(t_{3}+t_{4}\right)}{t}. \end{array}$$

In (5), variable *t* is the time required for waste collection from a fixed site per trip; *t*_3_ is the time required to drive from dispatch station to first container location to be served by the day; *t*_4_ is the time required to drive from the last container location to be served by the day to the dispatch station.

The time taken per trip is given by the expression shown below:

(6) $$\begin{array}{c} t_{5}=\frac{t_{6}-t}{Y}. \end{array}$$

In (6), variable *t*_6_ is the number of trips made in a week. The number of trips made per week is given by the expression shown below:

(7) $$\begin{array}{c} t_{6}=\frac{K}{RQ}. \end{array}$$

In (7), variable *K* is a measure of the volume of waste generated in a week in cubic meters per kg; *R* is the average size of the container in cubic meters per kg; *Q* is the weighted average container utilization factor.

Since *t* = (*t*_1_ + *t*_2_ + *Y*)/(1 − *A*) as shown above in (2), it follows that

(8) $$\begin{array}{c} t_{1}+t_{2}+Y=\left(1-A\right)t. \end{array}$$

Efficiency can thus be measured by the following relationship:

(9) E=Y+t1+t2+Btt=(t1+t2+Y)+Btt=(1−A)t+Btt=1−A+B.

Values of *E* in the expression *E* = 1 − *A* + *B* were calculated for each of the 1,034 businesses that took part in the study. Businesses for which values of *E* fell below the median were used for identifying businesses in which efficiency in waste management was inadequate. Businesses for which values of *E* were greater than or equal to the median were used for identifying businesses in which efficiency in waste management was deemed adequate. That is,

(10) $$\begin{array}{c} \text{overall}\;\text{efficiency}=\{\begin{array}{c} \text{inadequate}\;\;\text{if}\;\;\text{score}\;\text{is}\;\text{below}\\ \text{the}\;\text{median}\;\text{of}\;E\\ \text{adequate}\;\text{if}\;\text{score}\;\text{is}\;\text{greater}\;\text{than}\\ \text{or}\;\text{equal}\;\text{to}\;\text{the}\;\text{median}\;\text{of}\;E. \end{array} \end{array}$$

Pearson's chi-square tests of association [27] were used for the screening of variables. This was done by performing two-by-two tests of associations between overall efficiency in the management of waste and the various socioeconomic, demographic, sanitary, environmental, and health-related variables on which data was gathered as part of the study. At the 5% level of significance, an association was deemed significant if the *P* value was below 5%. The dependent variable of study is a measure of overall efficiency in the management of waste. The variable has only 2 possible values (Inadequate, Adequate). Since the dependent variable of study is dichotomous, binary logistic regression analysis [28] was used in order to identify key predictors of inefficiency in the management of waste. Multilevel analysis [29] was used in order to estimate the extent of variation with regards to efficiency in the management of waste by geographical zone and category of business enterprise. Multilevel analysis is a statistical procedure that enables planners and policy makers to allocate resources that are needed for intervention based on the extent of variation observed at various hierarchical levels. In this study, the two hierarchical levels depending on which efficiency in waste management varies are geographical location and category of business.

The degree of adherence to municipal bylaws and guidelines for the disposal of solid waste was measured by using a 2-point scale. The guidelines used for measuring adherence were the ISO 14000 and ISO 14031 guidelines for environmental management and performance monitoring in the management of waste and the environment [30–32]. At each of the 1,034 businesses selected for the study, the degree of adherence to municipal bylaws and procedures recommended for solid waste management by businesses by the city of Tshwane was graded based on ISO 14000 and ISO 14031 guidelines. That is, at each business enterprise, binary grades (Adequate, Not adequate) were allocated as a measure of compliance according to criteria stipulated in ISO 14000 and ISO 14031 guidelines. The binary variable created for assessing degree of adherence to ISO guidelines or municipal bylaws was used as one of the predictor variables of study. Category 1 of the binary variable represented inadequate adherence to ISO guidelines or municipal bylaws. Category 2 of the binary variable represented adequate adherence to ISO guidelines or municipal bylaws.

The purpose of conducting binary logistic regression analysis was to identify influential predictors of inefficiency in the management of waste among businesses operating in the city of Tshwane. The use of binary logistic regression analysis was appropriate as the dependent variable of study had only 2 possible values (1,  0). In binary logistic regression analysis, odds ratios were used as an epidemiological measure of effect. At the 5% level of significance, influential predictors of inefficiency in waste management are characterized by estimated odds ratios that differ from 1 significantly, *P*-values that are smaller than 0.05, and 95% confidence intervals of odds ratios that do not contain 1. The adequacy of the fitted logistic regression model was assessed by using standard diagnostic procedures such as the classification table, the Hosmer and Lemeshow goodness-of-fit test, receiver operating characteristics (ROC) plots, and sensitivity/specificity plots [28].

## 3. Results

Table 1 shows a comparison between businesses that were efficient with regards to solid waste management with those that were not. It can be seen from the table that 857 of the 1,034 businesses (83%) were efficient, while the remaining 177 businesses (17%) were inefficient. The table shows that a significant percentage of businesses located in the central and western parts of the city were inefficient, whereas businesses located in the eastern and northern parts of the city were by and large efficient. The table shows that 76% of operators who managed businesses that were efficient with regards to waste management have acquired formal education at college level or better. In general, businesses that are inefficient in the management of waste are relatively younger, poor in personal hygiene and cleanliness of premises, and are by and large commercial. The majority of old businesses (6 years or more) are efficient in waste management. Businesses that are operated by owners are more efficient in comparison with businesses that are operated by employed managers. Utilization of private contractors for waste removal and management, regular inspection of premises by municipal workers, familiarity with the South African White Paper on waste management, source reduction of waste, good perception on the benefits of proper waste management, and adherence to waste management regulations recommended by the municipality are much more common among businesses that are efficient in waste management.

Two-by-two Pearson chi-square tests of associations [27] was used for performing a preliminary screening of influential factors that were significantly associated with inefficient management of waste.  Table 2 shows a list of 15 factors that are significantly associated with poor or less than satisfactory waste disposal at the 0.001 level of significance. In each of the tests, the outcome variable of study, *Y*, was defined as follows:

(11) $$\begin{array}{c} \text{overall}\;\text{efficiency}=\{\begin{array}{c} \text{inadequate}\;\;\text{if}\;\;\text{score}\;\text{is}\;\text{below}\\ \text{the}\;\text{median}\;\text{of}\;E\\ \text{adequate}\;\text{if}\;\text{score}\;\text{is}\;\text{greater}\;\text{than}\\ \text{or}\;\text{equal}\;\text{to}\;\text{the}\;\text{median}\;\text{of}\;E. \end{array} \end{array}$$

At the 0.001 level of significance, significant associations are characterized by large observed chi-square values and *P*-values that are smaller than 0.001.  Table 2 provides a list of 15 variables that are significantly associated with inefficient waste management.

At the 0.001 level of significance, all 15 variables shown in Table 2 are significantly associated with overall efficiency in the management of waste. It can be seen from the table that the top 5 significant variables are: lack of adherence to municipal bylaws and regulations, wrong perception on the potential benefits of proper waste management, failure of businesses to provide customers with enough trash cans, the status of the business operator (owner or employee), and the frequency at which business premises are inspected by municipal sanitation and health workers, in a decreasing order of strength.

Results from binary logistic regression analysis are theoretically more reliable than results from Pearson's chi-square tests of association [28]. This is because the measure of effect in binary logistic regression is the odds ratio, and not two-by-two significant associations. Logistic regression analysis allows multivariate analysis involving several variables that are influential over waste disposal. It is also possible to assess the reliability of the fitted logistic regression model based on highly reliable diagnostic tests such as the classification table, the likelihood ratio test, the Hosmer-Lemeshow goodness-of-fit test, as well as receiver operating characteristic (ROC) and sensitivity/specificity plots [28].

At the 0.05 level of significance, influential predictor variables are characterized by estimated odds ratios that differ from 1 significantly, *P*-values that are smaller than 0.05, and 95% confidence intervals of odds ratios that do not contain 1. Accordingly, it can be seen from Table 3 that all 4 predictor variables are significant at the 0.05 level. The results show that efficiency in the proper management of solid waste is significantly influenced by 4 predictor variables. These 4 influential predictor variables are lack of adherence to municipal bylaws and regulations (OR = 9.17; 95% C.I. = (6.42, 12.54)), wrong perception (OR = 8.81; 95% C.I. = (6.01, 11.35)), failure to provide customers with enough trash cans (OR = 3.15; 95% C.I. = (1.46, 5.87)), and the operation of businesses by employed managers (OR = 2.69; 95% C.I. = (1.66, 4.32)), in a decreasing order of importance.

The adjusted odds ratio of the variable poor adherence is 9.17. This shows that a business that is managed by an operator who fails to adhere to guidelines set out for waste management by the city of Tshwane is 9.17 times as likely to be inefficient in the proper management of solid waste in comparison with a business that is managed by an operator who adheres to recommended guidelines. The adjusted odds ratio of the variable wrong perception is 8.81. This shows that a business operator who has the wrong perception on the benefits of proper management of solid waste is 8.81 times as likely to be inefficient in comparison with another business operator with the correct perception on the benefits of proper waste management. The adjusted odds ratio of the variable failure to provide customers with enough trash cans is 3.15. This shows that a business in which there are not enough trash cans for customers is 3.15 times as likely to be inefficient in the management of solid waste in comparison with a business in which enough trash cans are provided to customers. The adjusted odds ratio of the variable status of operator is 2.69. This shows that an outlet that is operated by someone who does not own the business being operated is 2.69 times as likely to beinefficient in the proper management of solid waste in comparison with an outlet that is operated by someone who actually owns the business. Adjusted odds ratios are more reliable than unadjusted odds ratios in epidemiological studies of this kind. In this study, the estimated odds ratios were adjusted for two potential confounding variables (level of education of business operator and the physical location of business). The adjusted odds ratios did not differ much from the unadjusted odds ratios, thereby showing that none of the variables used for adjustment was a confounding variable. There was no effect modifying variable.

### 3.1. Goodness-of-Fit Tests

**Figure 1:**
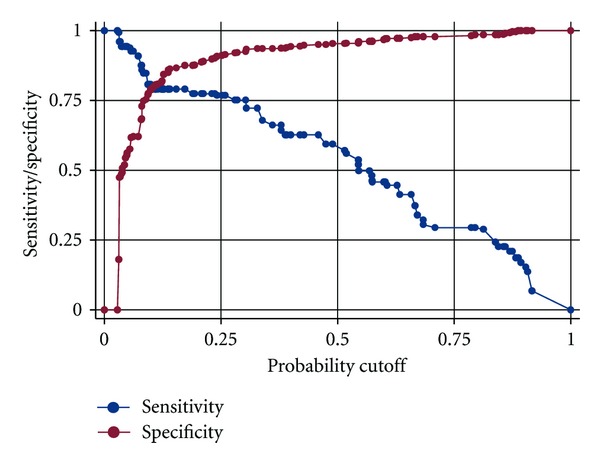
Plot of sensitivity/specificity versus probability cut-off point.

The reliability of the fitted logistic regression model was assessed using standard goodness-of-fit tests suitable for binary logistic regression analysis [28]. The classification table showed that the fitted model had an overall percentage of correct classification of 88.78%, a percentage sensitivity of 57.06%, and a percentage specificity of 95.33%. This shows that the fitted model is highly reliable in accurately classifying observations. The Hosmer-Lemeshow goodness-of-fit test gave a *P*-value of 0.0701, a figure which is greater than 0.05, thereby showing that there was no reason to doubt the reliability of the fitted logistic regression model.  Figure 1 below shows a plot of sensitivity/specificity versus probability cut-off point. The two plots cross each other fairly close to the vertical axis. This shows that the fitted model is adequately sensitive and specific.

**Figure 2:**
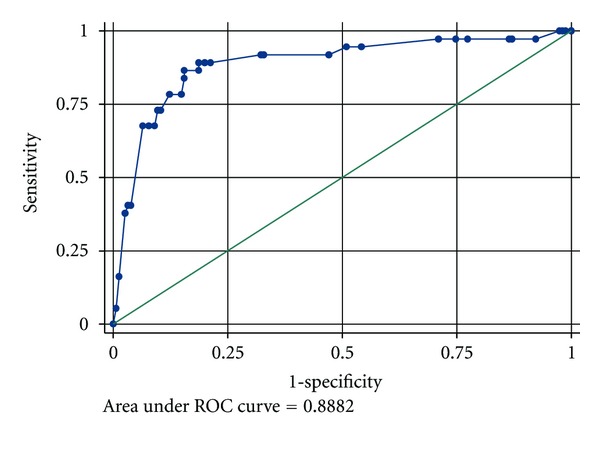
Area under the Receiver Operating Characteristic (ROC) plot.

Figure 2 below shows a Receiver Operating Characteristic (ROC) plot. The magnitude of the area that lies under the ROC plot is a measure of variation explained by the fitted logistic regression model. In this case, the area under the ROC plot is 88.82%, a figure that is significantly above 75%. The unexplained proportion of variation is equal to 11.18%. The large proportion of explained variation and the small proportion of unexplained variation show that the fitted model is highly reliable in explaining variability in waste disposal as a function of the explanatory variables used for logistic regression analysis.

The likelihood ratio test is used for assessing the collective efficiency of the 8 predictor variables used for performing binary logistic regression analysis. At the 5% level of significance, a *P*-value that is smaller than 0.05 shows that the 8 predictor variables used for performing binary logistic regression analysis are jointly efficient. In this case, the *P*-value from the likelihood ratio test is equal to 0.000, a figure that is smaller than 0.05. This small *P*-value shows that the 8 predictor variables used for binary logistic regression analysis are collectively efficient in accounting for failure in the proper disposal of waste.

Multilevel analysis [29] was used in order to estimate the extent of variation with regards to efficiency in the management of waste by geographical zone and category of business enterprise. Table 1 provides frequency distributions for each of the 7 categories of business (agricultural, commercial, construction, industrial, institutional, municipal, and processing and manufacturing) as well as 5 geographical locations (central, east, west, north, south) of the city of Tshwane that were considered in performing multilevel analysis. Results obtained from multilevel analysis showed that there were significant differences among the 7 categories of waste. The results showed that 23.05% of the total variation in efficiency is due to differences among the 7 categories of waste produced by business enterprises operating in the city of Tshwane. The results also showed that businesses within the same category of waste and geographical location were equally efficient in the management of solid waste.

## 4. Discussion

The key objective of research was to identify and quantify factors that affect efficiency in the management of solid waste by businesses that are operated in the city of Tshwane. The study has shown that efficiency in the management of solid waste is significantly influenced by 4 key predictor variables of study. These 4 influential predictor variables are lack of adherence to municipal bylaws and regulations, wrong perception on the potential benefits of proper waste management, failure to provide customers with enough trash cans for waste disposal, and the practice of operating businesses by employed managers, in a decreasing order of importance. In the city of Tshwane, municipal solid waste consists of everyday items such as product packaging, empty bottles and cans, grass clippings, furniture, clothing, left-over food, newspapers, appliances, paint, batteries, pieces of metals, and so forth. Such solid waste is generated by businesses, households, schools, hospitals, and visitors travelling into the city on foot or by other modes of transport such as cars, train, taxi, or bus. The Health Department of the city of Tshwane [2] has a municipal bylaw that stipulates how solid waste should be packaged, sorted, collected, and disposed of by inhabitants of the city. The bylaw encourages inhabitants of the city to practice source reduction of solid waste, recycling, and composting (collecting organic waste such as left-over food and garden refuse, storing these wastes under conditions that are designed to help them break down naturally, and then use the resulting compost as a natural fertilizer). According to the South African National Department of Environmental Affairs and Tourism [4], the disposal and combustion of municipal solid waste is conducted by the use of landfills, the conversion of non-recyclable waste materials into useable heat, electricity, or fuel, combustion, and transfer stations. Although the use of such mechanisms is consistent with the municipal bylaw in the city of Tshwane, the mechanisms have been poorly utilized mostly due to lack of infrastructural development and technical skills. 

Findings from this study are not surprising. The results are expected from a typical sub-Saharan African country in which poverty, unemployment, and massive immigration into urban centres prevail. Environmental hazards arising from the decomposition of solid waste under oversaturated conditions [33] and dumping of solid waste in illegal landfills often cause sludge [34]. Such problems are particularly evident in suburbs of Tshwane such as Mamelodi and Marabastad. In this regard, the city of Tshwane can be viewed as a combination of clean white suburbs and dirty black townships. Generally, awareness and regard for environmental sanitation are poor in black suburbs. The other key environmental hazard is caused by arbitrary landfill sites that dot the peripheries of the city of Tshwane. Green waste from landfills produces potentially harmful gasses such as methane and leachates. Such products pollute water reservoirs in the city. The study by Snyman and Vorster [17] has found that composting and the pretreatment of municipal waste before landfilling are viable options for the city of Tshwane. Silva et al. [35] as well as Wiszniowski et al. have found that composting and pretreatment of municipal waste before landfilling significantly reduce the volume of solid waste and contributes for overall environmental sanitation [36]. At the moment, the city of Tshwane does not have adequate capacity for large scale composting, and there is an acute need for addressing this shortcoming.

The study has shown that a combination of technical and administrative solutions is required in order to improve efficiency in the management of solid waste in the city of Tshwane. To separate waste generated at the various businesses, it is necessary to provide businesses with custom-made containers that are suitable. It is equally important to enforce municipal bylaws in order to ensure compliance. The frequency of collecting waste should be balanced with the volume of waste generated by the various businesses. Studies conducted in various parts of the developed world [37] have shown that there are economic benefits in outsourcing the collection and disposal of recyclable waste to the informal sector, and that sanitary landfills should be used for the final disposal of solid waste. In addition to enforcing the law with vigour, it is equally important to provide community based health education on environmental sanitation by collaborating with the Department of Health. Awareness campaigns and socioeconomic incentives could be provided by civic society and nongovernmental organizations that have a vested interest in improving environmental sanitation and cleanliness. Since most of the waste generated in the city of Tshwane is organic, it is recyclable. As such, the provision of incentives and education at the grass-roots level carries a clear socioeconomic merit. Composting is a form of aerobic treatment, and is suitable for treating organic waste in the city of Tshwane. According to Barlaz [38], facilities used for storage and collection of waste must be compatible with each other. Waste disposal and processing sites must be located strategically so that the cost of waste collection, disposal and processing becomes optimal. The city of Tshwane needs to make the necessary initial investment available in order to benefit from composting in the long-run. Extensive waste management research conducted in various parts of China [39] has shown that educating rural as well as urban people on how to produce compost by using low technology has long-term economic benefits to big metropolitan municipalities such as Tshwane. Large scale compost activities require massive infrastructural investment and skills based training. Research conducted in Nigeria [40] has shown that strategic partnerships and collaboration among academic and research institutions and municipalities have the potential for enhancing overall efficiency in waste management, skills development, and the creation of employment opportunities in municipalities such as Tshwane. One particular area of waste that stands to benefit out of such partnership is the management and processing of plastic and e-waste. This is because unprocessed plastic waste has almost no economic benefit. The collection and recycling of plastic waste is characterized by serious challenges and difficulties in the city of Tshwane. The city of Tshwane has no coherent policy on the collection of e-waste. Neither does it provide clear incentives to entrepreneurs who wish to collect, classify, and process e- and plastic waste. Since e-waste could be hazardous, there is a dire need to build capacity in the classification of e-waste into one of two categories (harmful or hazardous), and processing each category of waste by utilizing an appropriate form of technology. There is an acute need for providing incentives, enforcing municipal bylaws, the provision of health and sanitary education, and a comprehensive monitoring and evaluation programme for assessing the progress made in this regard regularly. A well-functioning integrated solid waste management system can only be realized in the city of Tshwane by providing clear incentives for good practice and behaviour, and by severely penalizing irresponsible behaviour in the city. Liu et al. [41] have found that the provision of direct socioeconomic incentives, clear guidelines on the collection, disposal, and processing of e- and plastic waste, as well as a strict enforcement of municipal bylaws is required for improving overall efficiency in the management of e and plastic waste in the city of Tshwane.

The efficient management of solid waste produced by enterprises that conduct business in the various parts of the city of Tshwane has numerous economic, sanitary, and health-related benefits to the inhabitants of the city. Disposing of waste in landfills is much better than using open dumps. Up until recently, emphasis has been placed on waste disposal, and not on management, recycling, and composting. Poor management of waste has an adverse impact on the environment and public health, particularly in townships such as Mamelodi, Marabastad, Soshanguve, and Attridgeville. In these townships, waste is managed poorly, and landfills are inappropriately sited, designed, managed, and operated. Until recently, the management of waste generated by businesses operating in the city has not been given due consideration. The waste management that took place focused mainly on waste disposal and was reactive in that it addressed needs as they occurred. Holistic, integrated waste management planning was poorly done. The low priority that was historically accorded to waste management has resulted in waste impacting detrimentally on the South African environment and on human health. Standards for medical waste incinerators are generally inadequate in comparison with international best practice.

Efficient waste disposal is a process that requires the full collaboration of all stakeholders on a community based collaborative approach. In addition to providing sanitary education and inspection services to the businesses, clear incentives must be provided to ensure maximum success. The enforcement of regulations, the provision of incentives, adequate logistical resources, additional manpower, financial rewards, public-private partnerships, and awareness campaigns are all essential.

The treatment of waste produced by the businesses in the city of Tshwane is similar to waste produced in a typical developing nation in the sense that treatment of this waste involves simply a reduction of its volume by use of methods such as baling or shredding although incineration and composting is practiced at a small scale. The emphasis remains on disposal of general waste by landfill without treatment as the lowest cost disposal option, as landfill airspace is still available in South Africa. The lack of pretreatment of general waste before disposal is therefore currently not regarded as a problem in South Africa. Incineration of general waste and hazardous waste is not acceptable to many stakeholders due to the poor operation of many existing facilities and noncompliance with existing by-laws. Incineration is not economically feasible in South Africa since its warm climate limits the market for the energy derived from the incineration process. The majority of operating incinerators in South Africa are used for the treatment of infectious medical waste.

Increasing general awareness about the benefits of proper waste disposal is a key requirement for success. The number of waste disposal sites is limited, and the disposal of waste is expensive. Since there are not enough of these facilities, hazardous waste is often transported over long distances, resulting in increased risks of accidents and higher transport costs. Some other helpful steps are to undertake an integrated plan in which waste is gathered and disposed of efficiently based on mutual collaboration among stakeholders, strengthening the technical, financial, administrative, and operating capacity of the institutions in the basic environmental sanitation sector, and encouraging health education and community promotion activities, which are basic to the success of waste collection and disposal, especially at taxi ranks and the streets at the central business district of Pretoria, to provide clear incentives to businesses that improve the quality of waste collection and disposal based on generally accepted standards. Technical cooperation among stakeholders must be directed toward the strengthening of institutions in the basic environmental sanitation sector and emphasis should be given to the following activities: operation and maintenance, community promotion, training, administration and management, the preparation of plans and studies helpful for efficient waste disposal, and the application of technologies that are helpful for efficient waste disposal.

The need for inspection becomes acute during rainy seasons and prolonged civil actions. In-depth interviews conducted with some of the managers of the 1,034 businesses that took part in the study have revealed that the businesses suffer enormously during prolonged strike actions. Waste material gets vandalized by scavengers as a result of delay in collection by service providers. It is too risky to leave waste uncollected during rainy seasons as waste could easily be mixed up with excreta that could be washed away by rain-water, ending up in wells and streams. The germs in the excreta could then easily contaminate drinking or washing water. In such situations, diarrhoeal diseases can spread from one person to another. Failure to dispose of waste can have a significant effect on the health of communities. Where refuse is not disposed of properly, it can lead to pollution of surface water, as rain washes refuse into rivers and streams. There may also be a significant risk of groundwater contamination. Refuse disposed of in storm drains may cause blockages and encourage fly and mosquito breeding. It is therefore very important that household waste is disposed of properly. All business premises operating in the city of Tshwane must be inspected for environmental sanitation and cleanliness regularly with a view to encourage and reward good practice, and to penalize irresponsible behaviour. Municipal bylaws on the collection, disposal and management of waste must be enforced with enough vigour and commitment. Failure to do so can easily result in frustration among businesses that obey municipal guidelines and regulations on waste management.

## 5. Conclusions

The study showed that 17% of the 1,034 businesses were not efficient enough with regards to the proper collection, disposal, and management of solid waste. The study has shown that efficiency in the management of solid waste is adversely affected by lack of adherence to municipal bylaws and regulations on proper waste management, wrong perception on the potential benefits of proper waste disposal, failure to provide customers with enough trash cans at business premises, and the operation of businesses by employed managers, in a decreasing order of importance. Lack of adherence to municipal bylaws and regulations that are essential for proper waste management constitutes a key challenge in the city of Tshwane. The presence of wrong perception on the potential benefits of waste disposal is also a well known hurdle. To rectify this issue, a combination of three interventions is necessary. The first intervention is to enforce municipal bylaws with vigour. The second intervention is to provide incentives to businesses that manage solid waste properly in accordance with guidelines provided by the city of Tshwane. Regulatory and legislative actions must be taken against those who fail to respect municipal bylaws that are related to cleanliness and proper waste management. The efficient disposal of waste generated by businesses operating in the city has direct economic benefits to all inhabitants of the city. Accordingly, waste should be gathered efficiently and disposed of in accordance with the waste collection and management plan produced by the city of Tshwane. Management of waste must start at the lowest level. The third intervention is to actively promote an education campaign in all parts of the city of Tshwane with a view to ensure the full collaboration of businesses conducting business in the city.

Based on findings of this particular study, the following recommendations are made to the city of Tshwane in order to improve overall efficiency in the management of solid waste that is generated by businesses operating in the city.

- The city of Tshwane must produce and implement an integrated plan for the management of solid waste in collaboration and partnership with the relevant stakeholders in the city so that each of the role players in the waste management chain can investment adequately in basic environmental sanitation.
- An initial infrastructural investment needs to be made by the city of Tshwane in order to build adequate capacity for commercial composting. This should be done in collaboration with business enterprises so that they can share the financial burden at the initial stage, and benefit from compost-related business opportunities in the long-run.
- The city of Tshwane must strive to increase awareness about the potential benefits of proper waste collection and disposal by promoting health education on environmental sanitation and techniques that are useful for collecting and sorting waste. Incentives must be provided to businesses that do a good job in terms of the proper collection and disposal of solid waste. The city must support community-based health promotion activities undertaken by nongovernmental organizations. The city must also support research initiatives conducted by academic and research institutions in areas that are related to waste management, environmental sanitation, and personal hygiene by funding them partially or fully.
- The city of Tshwane must improve the conditions of employment of municipal workers responsible for waste collection and disposal.
- The city of Tshwane and Gauteng Department of Health must provide technical assistance to businesses that do not have their own waste management plans so that such businesses can contribute for overall efficiency in environmental sanitation.

## Figures and Tables

**Table 1:** Comparison with regards to overall efficiency in waste management.

Characteristic	Efficient (*n* = 857)	Inefficient (*n* = 177)
	Agricultural: 1%	Agricultural: 1%
	Commercial: 71%	Commercial: 83%
	Construction: 6%	Construction: 3%
Category of business	Industrial: 11%	Industrial: 3%
	Institutional: 2%	Institutional: 3%
	Municipal: 6%	Municipal: 5%
	Manufacturing: 3%	Manufacturing: 2%

Geographical location of business in the city of Tshwane	Central: 31%	Central: 41%
	East: 21%	East: 13%
	West: 19%	West: 23%
	North: 17%	North: 13%
	South: 12%	South: 10%

Age of business in years	Less than a year: 5%	Less than a year: 34%
	1 to 2 years: 6%	1 to 2 years: 35%
	3 to 5 years: 29%	3 to 5 years: 15%
	6 years or more: 60%	6 years or more: 16%

Status of business operator	Owner: 76%	Owner: 31%
	Manager: 24%	Manager: 69%

Level of education of operator	College level or better: 76%	College level or better: 39%
	High school level or less: 24%	High school level or less: 61%

Gender of operator	Male: 76%	Male: 73%
	Female: 24%	Female: 27%

Use of private contractor for waste management	Yes: 23%	Yes: 16%
	No: 77%	No: 84%

Sorting waste	Yes: 76%	Yes: 45%
	No: 24%	No: 55%

Adherence to waste management regulations	Yes: 95%	Yes: 52%
	No: 5%	No: 48%

	Excellent: 9%	Excellent: 0%
	Very good: 43%	Very good: 21%
Personal hygiene	Satisfactory: 37%	Satisfactory: 41%
	Less than satisfactory: 10%	Less than satisfactory: 33%
	Poor: 1%	Poor: 5%

Perception on the benefits of proper waste management	Excellent: 3%	Excellent: 1%
	Very good: 56%	Very good: 6%
	Satisfactory: 35%	Satisfactory: 29%
	Less than satisfactory: 5%	Less than satisfactory: 55%
	Poor: 1%	Poor: 9%

Source reduction of waste	Yes: 80%	Yes: 52%
	No: 20%	No: 48%

Amount of waste generated in 1,000 kg per week	≤0.9 : 25%	≤0.9 : 49%
	1 to 1.9 : 46%	1 to 1.9 : 42%
	2 to 4.9 : 27%	2 to 4.9 : 8%
	5 to 9.9 : 2%	5 to 9.9 : 1%
	≥10 : 0%	≥10 : 0%

Enough trash cans available for customers	Yes: 77%	Yes: 46%
	No: 23%	No: 54%

Regular inspection of premises by municipality	Yes: 84%	Yes: 41%
	No: 16%	No: 59%

Familiarity of operator with White Paper on Waste Management	Yes: 86%	Yes: 28%
	No: 14%	No: 72%

**Table 2:** List of top 15 significant associations from Pearson's chi-square tests of associations with overall efficiency in waste disposal (*P* < 0.001).

Variable of study associated with overall efficiency in waste management	Observed chi-square value	*P* value
Adherence: degree of adherence to waste management regulations	716.04	0.0000
Perception: perception on the benefits of proper waste management	705.99	0.0000
Trashcan: availability of enough trash cans for customers	701.42	0.0000
Status: status of person operating business (owner or employee)	469.21	0.0000
Frequency: frequency at which business premises are inspected by municipality	299.57	0.0000
Hygiene: personal hygiene of employees at business premises	251.72	0.0000
Maintenance: degree of maintenance of trash bins and their environment in business premises	167.09	0.0000
Cleanliness: degree to which business premises are kept clean	139.88	0.0000
Education: level of education of business operator	127.52	0.0000
Inspection: regular inspection of premises by municipal workers	115.14	0.0000
Volume: volume of waste generated	109.59	0.0000
Contractor: use of private contractors for waste management	104.44	0.0000
White Paper: familiarity with White Paper on waste management	103.87	0.0000
Implement: degree to which a waste management plan is implemented	100.11	0.0000
Sort: sorting waste generated at source	93.12	0.0000

**Table 3:** Odds ratios estimated from binary logistic regression analysis.

Variable	Unadjusted OR and 95% C.I.^+^	*P* value	Adjusted* OR and 95% C.I.^+^
Poor adherence	9.18 (6.43, 12.55)	0.000	9.17 (6.42, 12.54)
Wrong perception	8.84 (6.02, 11.36)	0.000	8.81 (6.01, 11.35)
Failure to provide customers with enough trash cans	3.17 (1.48, 5.89)	0.000	3.15 (1.46, 5.87)
Status of operator (owner, manager)	2.71 (1.69, 4.35)	0.000	2.69 (1.66, 4.32)

*Adjustment was done for level of education, gender, and location of business.

^+^C.I.: Confidence interval.
